# Cell Fusion in Malignancy: A Cause or Consequence? A Provocateur or Cure?

**DOI:** 10.3390/cells8060587

**Published:** 2019-06-14

**Authors:** Jeffrey L. Platt, Marilia Cascalho

**Affiliations:** 1 Department of Surgery, University of Michigan, Ann Arbor, MI 48109, USA; marilia@umich.edu; 2 Department of Microbiology and Immunology, University of Michigan, Ann Arbor, MI 48109, USA

**Keywords:** cell fusion, malignant transformation, progression, tumor immunity, tumor resistance

## Abstract

Cell fusion has been observed in malignancy, and cancer cells have been found especially apt to fuse with other cells. Investigation of human and experimental malignancies suggests spontaneous fusion of normal cells can induce manifold genetic changes and manifestations of malignant transformation. Fusion of transformed cells with other cells can promote the progression of cancer to more malignant forms. However, observations in various fields suggest cell fusion also potentially contributes to natural defenses against cancer. Thus, cell fusion potentially corrects genetic and/or phenotypic changes underlying malignant transformation. Cell fusion also might help nonmalignant cells in tumors thwart tumor growth. Perhaps most importantly, cell fusion may generate genetic changes that lead to the expression of neoantigens, provide the mass of neoantigen expression needed to elicit immunity, and promote the function of antigen-presenting cells in a way that favors protective immunity as a defense against malignancy. To the extent that cell fusion promotes cellular, tissue, and/or systemic resistance to malignancy, the propensity of tumor cells to fuse with other cells might constitute a natural defense against malignancy.

## 1. Introduction

The impact of cell fusion on biology and genetics has been of a subject of interest for many years (see [1,2] for review). Cell fusion occurs in normal placenta, skeletal muscle, and bone [3,4,5]. Cell fusion also occurs in response to injury in liver, heart, and interstitial tissues; in various viral and bacterial infections; and, quite often, in cancer. Of the various conditions in which cell fusion is observed, none is more controversial and potentially more important than malignancy.

**Figure 1:**
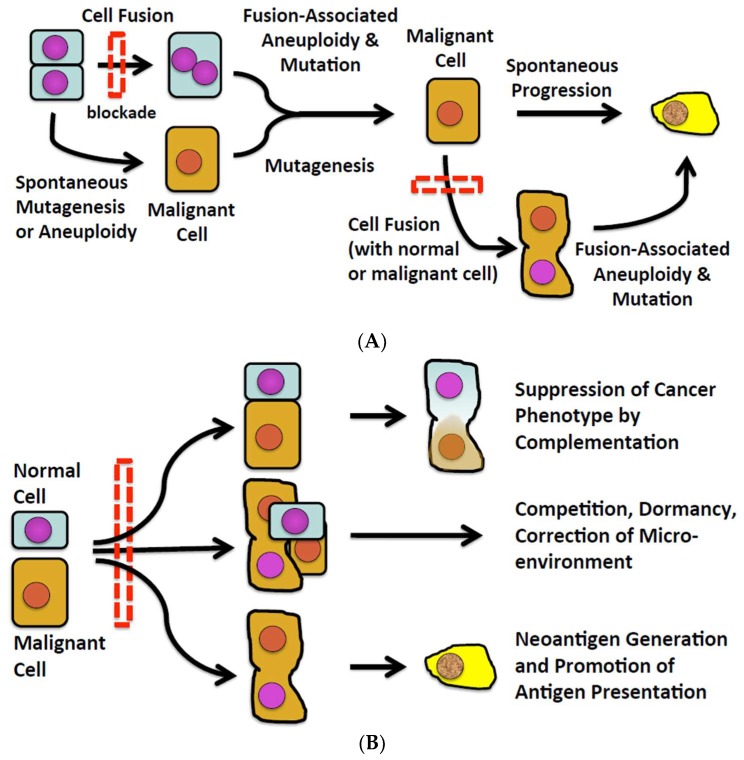
(**A**) The conventional view of cell fusion in malignancy. (**B**) A new perspective of cell fusion as a defense against malignancy.

Numerous observations in experimental animals and some human subjects suggest that fusion of one normal cell with another can induce malignant transformation, and fusion of a cancer cell with other cells can spark the progression of an existing to a more malignant phenotype (Figure 1A). However, there also exist observations in various and disparate fields of inquiry potentially connecting cell fusion with natural defenses against malignancy (Figure 1B). How cell fusion could pose a barrier to malignancy and progression of malignancy has not been systematically explored. We do so here.

## 2. Cell Fusion in Malignancy

Cell fusion has been detected in some human and animal malignancies (for review, see [2,6,7,8,9,10,11,12,13]), however the process of fusion is rarely, if ever, observed and therefore the prior fusion of a cell must be inferred, usually by analysis of karyotype or complementation in chimeric individuals. Fusion of cancer cells with normal cells in experimental systems can diversify the genome and phenotypes of malignant cells and promote progression [7,10,14,15,16,17,18,19,20]. How often cell fusion does so in naturally arising human malignancies is unknown because few, if any, markers besides the presence of multiple nuclei reliably establish a given cell to be a hybrid. Perhaps the most compelling evidence of hybridization of cancers in human subjects and experimental animals is found in hematopoietic chimeras. As one recent example, LaBerge et al. [21] reported that malignant mononuclear cells isolated from a melanoma and metastases of the melanoma in a bone marrow transplant recipient contained DNA from the bone marrow transplant donor and the recipient. Although the process leading to hybridization was not witnessed, the presence of substantial amounts of donor and recipient DNA in mononuclear hybrid cells most likely reflected spontaneous fusion of donor and recipient cells.

Thus, important questions concerning cell fusion in cancer have long persisted. These questions include: (i) how often cell fusion actually occurs at the inception or after development of cancer; (ii) whether cell fusion causes or results from malignant transformation; (iii) whether cell fusion promotes progression of malignancy or progression promotes cell fusion; (iv) whether cell fusion promotes resistance to malignant transformation or progression of malignancy; and (v) if cell fusion does protect against malignancy and/or progression, whether surviving hybrid tumor cells reflect the failure of protective mechanisms. These questions have been of theoretical interest for many years [1,14,22,23]. Inextricable from these questions are controversies about the mechanisms underlying genetic and chromosomal changes in cancer, especially whether malignancies arise by stepwise accumulation of point mutations or chromosomal damage, aneuploidy, and/or chromosomal instability [24].

These questions occurred to us as we considered the implications of our own work and work conducted by others over time. We found that deliberate fusion of normal epithelial cells can induce malignant transformation and progression of malignancy that ensues [24]. Because fused cells were cloned, it was possible to determine the frequency of transformation and progression in relation to the fusion event. On the other hand, when observed as a spontaneous event in a heterologous system, cell fusion occurred frequently but never eventuated in evident malignancy [25]. There are good reasons to propose that malignant transformation might have occurred in the system in which we observed spontaneous cell fusion (i.e., human hematopoietic stem cells engrafted in fetal swine). However, it is also possible that spontaneous fusion of cells induces protections as well as genetic changes and that the latter dominate in most instances. As we consider the existing set of clinical and experimental observations, it is evident that because fusion events cannot be witnessed, further investigation of human cancers and basic cell biology will offer only provisional answers to the questions posed above. However, the development and testing of agents that specifically and effectively block cell fusion could dramatically advance understanding and perhaps answer all of the questions of long standing about cell fusion in cancer. Therefore, as we discuss observations suggesting cell fusion could promote resistance to malignancy, we shall also consider how specific blockade of cell fusion would modify such resistance.

## 3. Cell Fusion Blockade

Therapeutic agents that specifically and effectively inhibit cell fusion do not as yet exist but are likely to emerge. Investigations connecting viral or cellular fusogens with cancers [4,26,27] make fusogens and related molecules attractive targets for therapeutics [28]. Reports connecting cell fusion with the inception and progression of malignancy, such as a report by Duelli et al. [29] using bulk populations of cells and our report on clonal populations [24], might be taken to suggest that inhibition of cell fusion would impact favorably. Consistent with that concept, blockade of IL-4 receptors, the ligation of which induces fusion of myoblasts, suppresses the initiation and progression of alveolar rhabdomyosarcoma [30], and digestion of syntaxin 1 with botulinum toxin C1 decreases progression of human glioblastoma cells in immunodeficient mice, as only two examples. While consistent with the importance of cell fusion in oncogenesis and tumor progression, the agents and approaches used to block cell fusion are not specific for the tumor cells (e.g., blockade of IL-4 receptors impacts many different cells and botulinum toxin digests proteins other than SNARE-type proteins) and the immunodeficiency of the hosts precludes the balancing impact on tumor immunity.

However, if highly specific agents were tested in physiologically normal systems, certain predictions could be proposed. If cell fusion occurs as a consequence of malignancy and/or progression, however, then inhibition of cell fusion should change neither the incidence nor the outcome of cancer. Of course, failure of a cell fusion inhibitor would not prove that cell fusion is a consequence of malignancy, as failure could also reflect the timing of delivery, the dose, or pharmacodynamic or pharmacokinetic limitations of the agent. Failure might also occur if fusion or progression proceeded via several independent pathways.

However, the test of a cell fusion inhibitor could yield another, more interesting result—an increase in the incidence or severity of a malignancy. Thus, while cell fusion has been long considered a potential mechanism of genetic change in malignancy, it has also been found to correct pathogenic changes (see [22,31] for review). Therefore, despite our own research and speculation focusing on pathogenic consequences of cell fusion [3,24,32], we think it prudent to consider whether and how the inhibition of cell fusion might weigh unfavorably in the outcomes of cancer. In doing so, we shall assume that a blocking agent is manifestly effective in vivo and put aside considerations of timing, dose, pharmacokinetics, and pharmacodynamics.

## 4. Genetic and Chromosomal Changes in Cancer

The evolution of multicellularity was associated with, if not dependent on, the evolution of cellular defenses against accumulation of mutations that could spark malignancy [33,34,35]. The evolutionary imperatives included the large number of cell divisions needed to establish the body-plan and sustain tissues, such as intestine and skin, and rapid cell turnover, perhaps in part to limit cumulative exposure to environmental mutagens during longer lifespans. These imperatives presumably selected for stringent and redundant governance of cell proliferation, telomere length, DNA synthesis and repair, and tumor suppression when controls fail [35,36,37]. The specific steps in evolution can be difficult or impossible to prove, but the “selective pressure” can be surmised by comparing risks and adaptations in larger mammals with those of smaller mammals [38]. As Abegglen et al. [36] determined, wild mice, with body mass of ~50 g and lifespan of 4.5 years, and humans should have a vastly lower risk of malignancy than elephants, which reach ~4800 kg body mass and live ~65 years. However, autopsy and necropsy results reveal a similar prevalence of cancers. One explanation sometimes offered is that the evolution of larger body mass and longevity was made possible by evolutionary changes in tumor suppression. Indeed, elephants have ~19 copies of *TP53*, the product of which arrests cell cycling and induces senescence and apoptosis [39], whereas humans and mice have one copy per haplotype [36,40]. However, given the fecundity and long evolutionary history of rodents, one might as well question why mice do not have more copies of *tp53* and as high a prevalence of cancer as humans. The hypothesis we explore is that events, such as fusion, that induce or closely follow upon malignant transformation and progression of cancer could have been appropriated by evolution for defenses against cancer.

## 5. Restoring Tumor Suppressor Gene Functions

The first suggestion that certain genes protect normal cells from malignant transformation emerged from experiments in which normal proliferating cells were deliberately fused with malignant cells [39,41,42]. In 1969, Henry Harris reported that fusion of normal murine fibroblasts with various lines of malignant murine cells led to the formation of stable hybrids that had chromosomal markers of both parental cell lines and did not form tumors in histocompatible mice [31,43]. Reversion of malignant phenotype to normal after fusion of malignant cells with normal cells was soon confirmed using human cells [44]. The absence of tumors in mouse and human hybrids was striking since the malignant parental cells always formed tumors.

As exciting and provocative as the observations of Harris were at that time, it was as apparent then as it is today that malignancy could not be addressed by deliberately fusing normal cells with cancer cells [32]. Rather, Harris drew insights from this model that eventually would transform understanding of malignant transformation and offer clues to potential consequences of the blockade of cell fusion. Thus, Harris also observed that tumor cell-normal cell hybrids occasionally regained the capacity to form tumors. Tumor cell-normal cell hybrids that initially failed to form tumors but reacquired malignancy appeared to have lost chromosomal segments that had originated from the normal parental cells [43]. Harris reasoned that the deleted chromosomal segments included tumor suppressor genes [31]. Harris’s observations thus prompt consideration of the possibility that blocking cell fusion could increase the incidence of de novo malignancy or make existing malignancies worse rather than better.

## 6. Malignancy in the Face of Tumor Suppression

Our own experience, however, appears to contradict the observations and conclusions one might take from the work of Harris. We conducted experiments designed to determine whether fusion of normal epithelial cells could initiate malignancy [24]. Rat epithelial cells that were manifestly not transformed, had a stable diploid karyotype, and never formed tumors in immunodeficient mice were fused using polyethylene glycol and then cloned. Clones generated from the fused cells frequently exhibited chromosomal instability and aneuploidy, a transformed phenotype, and the capacity to form tumors in immunodeficient mice, consistent with the observations of Harris [43] and others (see [45] for review). Clones that had not fused exhibited none of the features of transformed cells and never initiated tumors in immunodeficient mice. Of note was that aberrant chromosomal numbers or features in a given clone either persisted with little change or reverted toward diploidy, which is to say the propensity for chromosomal damage, translocation, and/or separation in mitosis was transient. We also observed that *TP53* in the hybrid clones tested retained wild-type sequence. Since the cells that gave rise to malignancy were cloned after fusion, it is unlikely that wild-type *TP53* was generated by reversion, and the results suggest that cell fusion can induce malignancy despite intact tumor suppression pathways. Thus, our findings (and other work) suggest malignant transformation potentially can bypass tumor suppression processes intrinsic to the cell.

## 7. Tissue-Level Defenses—Fibroblasts as an Example

Fortunately, intrinsic cellular defenses against transformation are not the only barriers to the development of malignancy. Tissue-specific defenses may explain profound variation in the incidence of malignancy in various tissues with rapid cellular turnover (e.g., small intestine has numerous, rapidly turning over cells but few malignancies compared with colon, in which cellular turnover is slower [46,47]). Tissue-level defenses may help explain the extent of protection against malignancy evident in large mammals (humans have 10^3^-fold more cells and live 30-fold as long as mice but have about the same risk of malignancy) [48,49,50]. Indeed, realization that cellular protections against mutagenesis might not suffice to prevent malignancy in tissues such as skin and intestine led to the concept of tissue-level defenses [51]. Below, we discuss how cell fusion, as one potential tissue-level defense, might suppress the initiation and progression of malignancy.

Recent years have brought increasing appreciation of the fact that nonmalignant cells associated with malignancies profoundly influence tumor growth and progression. Tissue-level defenses, such as contact inhibition, population maintenance, and the partitioning of stem cells and chromosomal DNA in ways that minimize accumulation of DNA copying errors, were therefore proposed [52] and confirmed, at least provisionally [53]. Endothelial cells, mesenchymal cells, and fibroblasts can secrete growth factors and sculpt a microenvironment with the requisite plasticity to support the enlarging tumor mass, with an extracellular matrix that concentrates growth factors that support tumor cell survival and proliferation. Nonmalignant cells intrinsic to tumors also help restrain or block extrinsic defenses such as tumor immunity. The trophic actions of these cells, such as activated fibroblasts, have been extensively explored and reviewed (see [54,55,56,57,58,59] for review). Investigation of nonmalignant cells intrinsic to grafts has also revealed that normal cells such as fibroblasts also potentially constrain tumor growth and progression [56,58,60].

What causes nonmalignant cells in tumors to decisively restrain rather than promote tumor growth is not clear. However, fusion of fibroblasts with tumor cells can govern how fibroblasts influence the biology of tumors [60,61,62,63,64]. As one particularly dramatic example, breast cancer cells have been found potentially to engulf and thus effectively fuse with normal mesenchymal cells [65] or fibroblasts [64], and in so doing, to form a dormant niche that could survive without expanding or progressing. While the dormant niche focally controls tumor growth, it also can generate a microenvironment and growth factors that can support robust growth of tumor cells [63,66]. Although these interactions, like the interactions first described by Harris [31], can generate acute changes in the behavior of cancer cells (i.e., dormancy or progression), the more important impact probably depends on longer-term changes in the extracellular matrix, growth factors, and metabolites that the dormant or hybrid cells generate and visit upon neighboring tumor cells. From this perspective, it seems reasonable to postulate that a cell fusion blocker might exhibit dramatically different short- and long-term efficacy or that the in vitro impact of cell fusion blockade might fail entirely to predict in vivo efficacy.

## 8. Extrinsic Defenses Against Malignancy—Tumor Immunity

For more than a century, the possibility that cancer might evoke immune responses which limit tumor growth and occasionally mediate spontaneous cure has drawn profound interest and sparked some controversy (see [67,68,69,70,71,72,73] for examples). The controversies of longest standing concern such matters as the relevance of transplantable tumors as models for spontaneous tumors, how often tumors arise and resolve spontaneously without clinical diagnosis, how often naturally evoked tumor immunity actually modifies the course of malignancies, and whether immunity could sometimes initiate malignant transformation and progression [67,74,75,76]. Controversies about the impact of immunity on the biology and outcome of cancer are addressed at least in part by the “cancer immunoediting” model [72,73,77,78,79]. Whether or not and how often this model faithfully represents the relationship between the immune system and malignancy over time, the model nonetheless provides a valuable framework for examining how cell fusion and blockade of cell fusion could impact immune-mediated defenses against malignancy. In our discussion of this subject, we deliberately put aside virus-induced cell fusion, as the topic has been thoroughly explored [29,80] and immunity to viral antigens potentially eclipses immunity to tumor antigens.

The cancer immunoediting model envisions a dynamic relationship between the immune system and cancer that evolves over three epochs (see [73,78,79,81] for detailed review). In the first epoch, malignant transformation, presumably owing to mutation and recombination, generates a tumor that may be more or less immunogenic and more or less diverse. Immunity evoked by protein variants may control the tumor fully, in which case spontaneous resolution likely precludes diagnosis, or immunity destroys tumor cells expressing many of the variants but tumor cells expressing others survive. A second epoch, representing dormancy, may ensue. In this period, the immune system and the tumor adapt to each other and the repertoire of tumor cells that survived the first epoch is “sculpted” to select cells expressing variants least recognizable by effector cells and/or to enforce tumor-induced immunosuppression. We should add that a condition we first described in transplantation and named “accommodation” to reflect acquired resistance to immune or inflammatory injury probably occurs in the first and/or second epoch and renders tumor cells inured to immune-mediated cytotoxicity [82,83,84]. In the third epoch, ongoing diversification and accommodation generates tumor cells that escape control by innate and adaptive immunity, chemotherapy, and so forth, and progression is manifest.

If cell fusion were to occur during the various stages of cancer immunoediting, otherwise enigmatic steps in the model might be explained. For example, cell fusion potentially causes the nearly instantaneous diversification of tumor cells needed to explain selection of escape variants. Thus, if one assumes that neoantigens arise only during accretion of point mutations [81,85], several challenging predictions follow. First, the number of cell divisions needed to establish a tissue and/or total accrued mutations should predict cancer development and immunogenicity (or susceptibility to checkpoint blockade or tolerance). While some observations may support these predictions [86], albeit not without dispute (as we discuss elsewhere [32]), there is little consensus about how to define immunogenicity and no evident correlation with organ size or cell turnover [87]. Even more concerning is that rapidly mutating cells might change antigen composition too quickly to enable generation of clones of sufficient size to release the minimal amount of antigen needed to evoke immunity and hence effective censoring. This problem is shared by rapidly mutating viruses such as HIV, which can be quite immunogenic but not subject to immune control [88]. While the minimal size of a mutation-bearing clone to evoke immunity is unknown, the size probably exceeds the approximate minimum of 1000 dendritic cells needed to evoke immunity to a surplus of defined peptide or cellular antigen [89,90]. If tumor progression is associated with more rapid diversification, this problem is worse. More slowly mutating tumors and dormant tumors, on the other hand, are confronted by a TCR repertoire that is ever changing, as ~1 million new T cells with distinct antigen receptors emerge from the thymus each day. These challenges are not unique to tumor cells. T and B cells undergo recombination, junctional diversification, and point mutation as antigen receptor genes are established and these undoubtedly include neoepitopes. Other, normal tissues also accumulate substantial numbers of mutations [46,91], yet the tissues express normal levels of MHC. Thus, if tumors induce immunoediting, normal cells must do so as well, and immunoediting, as recently depicted, selects from slowly diversifying tumor and nonmalignant cells on one side and T cells on the other.

Introducing cell fusion as a mechanism of clonal diversification and antigen presentation solves certain problems intrinsic to the immunoediting model. If cell fusion initiates malignancy, manifold chromosomal changes and mutations occur simultaneously and the diverse malignant clones may remain genetically stable long enough to generate the amounts of neoantigen needed to evoke immunity before further rounds of diversification occur [24]. Fusion of malignant cells also generates ancillary signals needed to recruit and activate antigen-presenting cells. Most important, however, may be that fusion of mutation-bearing cells with dendritic cells or other APC [4] engenders robust protective immunity [92,93]. Thus, cell fusion potentially enables or amplifies the early events predicted by the immunoediting model. Clearly, then, if blockade of cell fusion prevents the inception of immunity in some cases, it might also disrupt extrinsic defense against malignancy early in its course. What balance would be struck between these opposing processes is of course yet unknown.

Modification of cell fusion also potentially impacts in complex ways the extrinsic control of tumor dormancy and progression. Above, we discussed evidence that fusion of tumor cells with nonmalignant cells can favor dormancy, but we must add here the possibility that fusion of tumor cells with dendritic cells helps to prevent escape from dormancy. In either case, blockade of cell fusion could favor tumor escape. However, cell fusion also is connected with tumor progression and presumably with escape from dormancy, and accordingly, blockade of cell fusion could prevent or slow pathogenic consequences of cancer. Clearly, the investigation of tumor biology using tumor growth and metastasis as an endpoint, while essential for some purposes, fails to depict the complex processes at hand. Regardless of the validity of the immunoediting model, we think analysis of mutational complexity, including neoepitope frequency and tumor-specific T-cell responses [94] as they evolve over time, will offer a more complete picture of the biology of malignancies. Among the insights such analysis might yield are clues about when tumor cell fusion occurs, whether fusion is closely followed by immunity to neoantigens, and how cell-mediated immunity is censored.

## 9. Concluding Remarks

Like many others, we have thought of cell fusion as a process that potentially contributes to the inception and progression of cancer [3,4,24]. Conditions that cause cells to fuse, such as infection with oncogenic viruses and chronic inflammation, also cause cancer to develop. Experimental and clinical investigations reveal a clear correlation between cell fusion and cancer progression. Still, we would be remiss if we failed to consider observations connecting cell fusion with natural defenses against malignancy.

Given current knowledge and technologies, it is difficult, if not impossible, to weigh the extent to which cell fusion lowers or raises the barriers to malignancy and progression of malignancy. We have sophisticated tools for detecting malignancies, but we have no reliable tools for determining how many malignancies that should develop do not do so or how many malignancies arose and spontaneously regressed before detection occurred. The potential impact of cell fusion on progression may be more amenable to investigation, since rate of tumor growth, among other indices, can be measured. However, many variables govern the rate of tumor growth and processes such as cell fusion influence many variables. Therefore, if blockade of cell fusion were to become possible and were observed to slow progression of one tumor, we would not be surprised to find that blockade hastened the growth of another tumor. Further, if cell fusion influences immunity, we would not be surprised to find that sometimes blockade slowed and sometimes it hastened tumor growth. Still, if cell fusion is eventually found to be common in many types of malignancy, we would be tempted to propose a priori that expression of the fusion proteins and cytokines and the metabolic changes that in concert promote cell fusion constitute natural multifaceted defenses against development and/or expansion of malignancy and that the tumors we observe represent the occasional failure of those defenses.
